# Pulmonary Interstitial Matrix and Lung Fluid Balance From Normal to the Acutely Injured Lung

**DOI:** 10.3389/fphys.2021.781874

**Published:** 2021-12-20

**Authors:** Egidio Beretta, Francesco Romanò, Giulio Sancini, James B. Grotberg, Gary F. Nieman, Giuseppe Miserocchi

**Affiliations:** ^1^Department of Medicine and Surgery, School of Medicine and Surgery, Università degli Studi di Milano-Bicocca, Monza, Italy; ^2^Univ. Lille, CNRS, ONERA, Arts et Métiers, Centrale Lille, FRE 2017-LMFL-Laboratoire de Mécanique des Fluides de Lille – Kampé de Fériet, Lille, France; ^3^Department of Biomedical Engineering, University of Michigan, Ann Arbor, MI, United States; ^4^Department of Surgery, State University of New York Upstate Medical University, Syracuse, NY, United States

**Keywords:** SARS-CoV-2, ARDS, edema, alveolar pressure, P-SILI, computational model

## Abstract

This review analyses the mechanisms by which lung fluid balance is strictly controlled in the air-blood barrier (ABB). Relatively large trans-endothelial and trans-epithelial Starling pressure gradients result in a minimal flow across the ABB thanks to low microvascular permeability aided by the macromolecular structure of the interstitial matrix. These edema safety factors are lost when the integrity of the interstitial matrix is damaged. The result is that small Starling pressure gradients, acting on a progressively expanding alveolar barrier with high permeability, generate a high transvascular flow that causes alveolar flooding in minutes. We modeled the trans-endothelial and trans-epithelial Starling pressure gradients under control conditions, as well as under increasing alveolar pressure (Palv) conditions of up to 25 cmH_2_O. We referred to the wet-to-dry weight (W/D) ratio, a specific index of lung water balance, to be correlated with the functional state of the interstitial structure. W/D averages ∼5 in control and might increase by up to ∼9 in severe edema, corresponding to ∼70% loss in the integrity of the native matrix. Factors buffering edemagenic conditions include: (i) an interstitial capacity for fluid accumulation located in the thick portion of ABB, (ii) the increase in interstitial pressure due to water binding by hyaluronan (the “safety factor” opposing the filtration gradient), and (iii) increased lymphatic flow. Inflammatory factors causing lung tissue damage include those of bacterial/viral and those of sterile nature. Production of reactive oxygen species (ROS) during hypoxia or hyperoxia, or excessive parenchymal stress/strain [lung overdistension caused by patient self-induced lung injury (P-SILI)] can all cause excessive inflammation. We discuss the heterogeneity of intrapulmonary distribution of W/D ratios. A W/D ∼6.5 has been identified as being critical for the transition to severe edema formation. Increasing Palv for W/D > 6.5, both trans-endothelial and trans-epithelial gradients favor filtration leading to alveolar flooding. Neither CT scan nor ultrasound can identify this initial level of lung fluid balance perturbation. A suggestion is put forward to identify a non-invasive tool to detect the earliest stages of perturbation of lung fluid balance before the condition becomes life-threatening.

## Introduction

Efficient gas diffusion in the air-blood barrier is only possible by strict control of extravascular water volume to prevent edema-induced tissue swelling and alveolar flooding. In normal lungs, the thickness of the air-blood barrier does not exceed ∼0.2 μm. Edema is prevented by an extremely low microvascular permeability and dynamic remodeling of the interstitial matrix (Miserocchi and Rivolta, 2012). The physiologic parameters controlling lung fluid balance are dynamic and change over the life span of individuals to ensure a healthy match of regional ventilation to the metabolic requirement.

This study merges the understanding of normal physiologic fluid balance control with the ramifications of what can occur when this control is lost. We will first review how a healthy lung can tightly control extravascular lung water by specific mechanical alterations to parenchymal structure that will efficiently minimize edema. Next, we will investigate how progressive loss of this anti-edemagenic control inevitably leads to severe edema. We present a detailed analysis of Starling pressure gradients sustaining flows across the endothelial and epithelial barriers. We stress the point that the heterogeneity in edema distribution allows us to distinguish between lung regions that can still provide control of fluid balance to allow gas exchanges and other regions that have lost this capacity because of breakage of the interstitial matrix. We correlate the Starling pressure gradients with the specific regional edema formation measured as the lung tissue wet-to-dry weight ratio (W/D). This study provides contribution to the understanding of acute lung injury pathophysiology and the role of mechanical ventilation in edema formation. In particular, this study is one of the first to discuss trans-endothelial and trans-epithelial flows as distinct events for a given lung distension pressure. It is hoped that this review might provide indications aimed at avoiding an unintentional ventilator-induced lung injury (VILI).

## The Air-Blood Barrier and the Molecular Structure of the Interstitial Compartment

The air–blood barrier (ABB) represents the interface for gas exchanges; the capillary endothelium is in close apposition with the alveolar epithelium so that the thickness of ABB is as low as 0.3 μm and its surface is in the range of 100 m^2^ in the human lung (Weibel, 2015).

**FIGURE 1:**
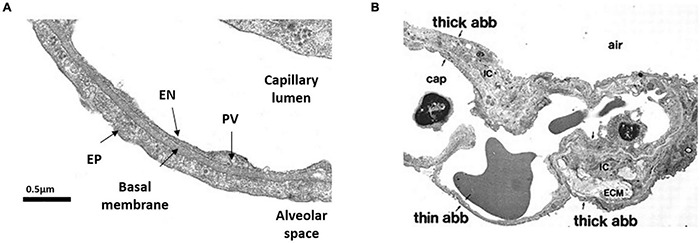
Morphology of the air-blood barrier (ABB). **(A)** Thin portion of ABB showing the close apposition between alveolar epithelium (EP) and capillary endothelium (EN). PV is plasmalemmal vesicle (from Botto et al., 2006). **(B)** Microscopy image showing the ABB and its thin and thick portions. ECM, extra cellular matrix; cap, lung capillary; ic, interstitial cell (from Conforti et al., 2002).

The ABB includes two portions: a thin side (Figure 1A) that accounts for more than 50% of alveolar surface that contains minimal interstitial space (Weibel, 2017), and a thick side (Figure 1B) that includes most of the parenchymal extra cellular matrix (ECM) and lymphatics. It has been suggested that the thick portion acts as a reservoir capacity where fluid filtered at the level of the thin portion might accumulate to be drained down a pressure gradient generated by lymphatics (Unruh et al., 1984; Conforti et al., 2002).

**FIGURE 2:**
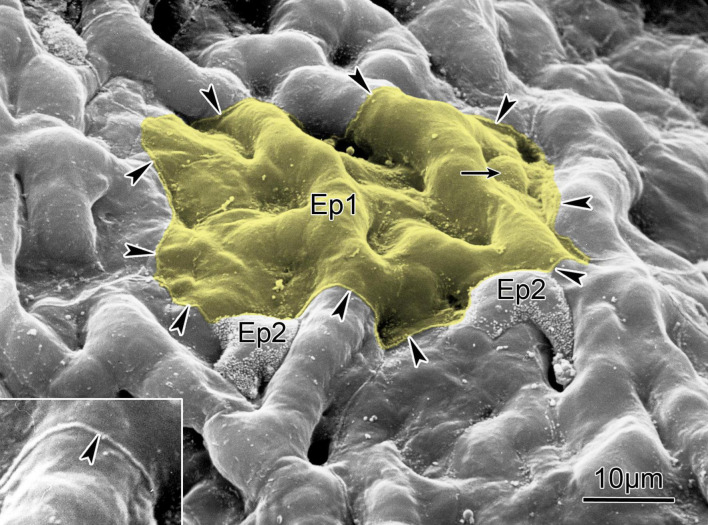
Scanning electron micrograph of the alveolar surface of a human lung showing protruding capillaries and two type II cells (Ep2) sitting in niches and characterized by a rim of microvilli. Small arrow points to cell body of a type I cell (Ep1) that covers several capillary meshes (yellow); the boundary of its cytoplasmic leaflet is marked by arrowheads outlining a small lip of the cell junction between adjoining cells (inset, compare Figure 1B). The surface area covered by this cytoplasmic leaflet is 1,300 mm^2^. Scale bar = 10 μm (from Weibel, 2015); reprinted with permission of the American Thoracic Society.

The special arrangement of the endothelial and epithelial cells is seen in Figure 2. Type-1 epithelial (Epi1) cells cover about 95% of the surface of each alveolus, with the rest being covered by globular Epi2 cells.

The body of Epi1 cells forms a series of interconnected structures that envelop, through several stalks, approximately 40 capillaries. There are only about 40 Epi1 cells lining a single alveolus facing about 170 endothelial cells, so any Epi1 cell covers about four endothelial cells. An average volume of ∼1,700 μm^3^ of an Epi1 cell spreads over a surface area of ∼5,000 μm^2^, and allows for a thickness of as low as 0.3 μm. Such a low number results from the combination of low number of Epi1 cells and extended plasma membrane surface. On the whole, the overall surfaces of Epi1 and endothelial cells are comparable.

Two important functional features of ABB are the following: very strict control of water balance by minimizing fluid exchanges across the endothelial and epithelial barriers, and remarkable mechanical resistance to the increase in tension related to change in lung volumes. These two important features rely on the structure-function relationship between the epithelial and endothelial cells and the macromolecular structure of the interstitial compartment separating the epithelial and endothelial layers.

The structure of the interstitial compartment includes a mesh of collagen and elastic fibers whose main role is to provide mechanical support and elasticity (Miserocchi and Rivolta, 2012). These molecules are chemically very stable and resistant to proteolysis. Within the space between these molecules, there is a dense mesh of molecules, belonging to the proteoglycan family, whose main role is to exert a strict control on the amount of extravascular water that must be kept to a minimum. Microvascular permeability is controlled by the low molecular weight heparan-sulfate proteoglycan (HS-PG, 300–500 kDa) of the basement membrane and by small peptidoglycans of the glycocalyx (PDGL), normally assuring high resistivity of the paracellular route (Reitsma et al., 2007). Large molecular weight chondroitin-sulfate proteoglycans (CS-PG > 1,000 kDa) bound to hyaluronan act as link proteins through low energy non-covalent bonds with other molecules and cells providing rigidity to the parenchymal mesh.

## The Control of Extravascular Water Under Physiological Conditions

The hydraulic pressure (Pint) in lung interstitial compartment is in the sub-atmospheric range, averaging ∼10 cmH_2_O (Miserocchi et al., 1990), and a negative Pint value represents the fluid dynamic equilibrium between capillary filtration down a high resistance pathway (low microvascular permeability) and a lower resistance pathway represented by lymphatic drainage. The equilibrium is also favored by the transcapillary oncotic pressure gradient (favoring absorption) that exceeds the hydraulic gradient (favoring filtration) (Parker et al., 1978).

**FIGURE 3:**
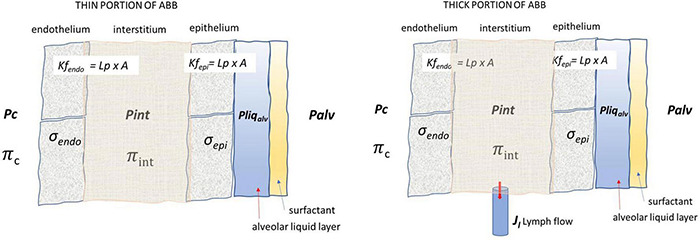
Lung fluid compartments of the ABB and parameters governing fluid exchanges. Pc, Pint, and Pliq_*alv*_ are capillary, interstitial, and alveolar liquid hydraulic pressure, respectively; Πc and Πint are capillary and interstitial oncotic pressure, respectively; σ_*endo*_ and σ_*epi*_ are the protein reflection coefficient of endothelium and epithelium, respectively; Palv is the alveolar pressure; Lp, A, Kf_*endo*_, and Kf_*epi*_ are the hydraulic conductance, overall filtration surface, and endothelial and epithelial filtration coefficient, respectively. Lymphatic drainage (Jl) occurs from the thick portion of ABB where lymphatics are located.

Figure 3 presents the fluid exchange models for the thin the thick portions of the ABB, and the parameters defining the pressure gradients that sustain potential water and solute fluxes across the endothelium and the epithelium. Note that lymphatics are only present in the thick portion of the ABB.

Transcapillary and transepithelial water exchanges are governed by the Starling law where *P* and Π are the hydraulic and the colloid osmotic pressures across any two compartments. Water flow (*J*_*v*_) is defined as:


(1) $$J_{v}=Kf\cdot \left[\left(P_{1}-P_{2}\right)-\sigma \left({\text{Π}}_{1}-{\text{Π}}_{2}\right)\right]$$

where *Kf* (*filtration coefficient*) = *Lp* ⋅ *A*, with *Lp* being the hydraulic conductance, *A* the surface area available for flow, and [(*P*_1_−*P*_2_)−σ(Π_1_−Π_2_)] the Starling pressure gradient generating flow.

In this study, we will focus on the Starling pressure gradients, and based on experimentally measured values, will analyze how they are modified to cause severe perturbation in lung fluid balance. Relevance will also be given to the estimate of the effect of increasing alveolar pressure on these gradients, a point that, so far, has remained unexplored but of utmost importance in mechanical ventilation. In a companion study, the Starling pressure gradients presented in this study will be used to model the transcapillary, transepithelial, and lymphatic flows in physiological condition as well as in developing severe lung edema.

Our whole analysis considers W/D as the more precise and reliable index of the lung fluid balance that has been related to experimentally measured values of variables and coefficients appearing in Eq. 1. The validity of W/D as an index of water balance was confirmed by Parker and Townsley (2004) who reported its excellent correlation with albumin and total proteins collected from the bronchoalveolar lavage fluid, an index of derangement of fluid balance in experimental models of graded lung injury.

Table 1 reports the Starling pressure gradients in a healthy lung at end-expiration and at end- inspiration. End-expiration corresponds to functional residual capacity (FRC) ∼22% TLC in supine position, while end-inspiration corresponds to ∼32% TLC (Agostoni and Mead, 1964). Water balance under physiological condition is defined by a lung wet weight/dry weight ratio (W/D) of ∼5 (Conforti et al., 2002). In Table 1 and from here on, positive values of a Starling gradient across the endothelium and the epithelium indicate fluid filtration into the interstitial space and the alveolar compartment, respectively; negative values of a Starling gradient indicate fluid clearance from these compartments.

**TABLE 1:** Trans-endothelial and trans-epithelial Starling pressure gradients at end-expiration (functional residual capacity, FRC) and end-inspiration under physiological conditions (referring to Point A in Figure 4).

	Trans-endothelial			Trans-epithelial	
	End-expiration	End-inspiration		End-expiration	End-inspiration
Pcap*	9	9	Pint**	−10	−24
Pint**	−10	−24	Pliq alv^#^	∼ 0	∼ 0
σ end^##^	0.85	0.85	σ epi^##^	0.85	0.85
Πcap**	26.8	26.8	Πint**	13.8	13.8
Πint**	13.8	13.8	Πliq alv	0	0
			γ	1	1
**ΔP**	**19**	**33**	**ΔP**	−**10**	−**24**
**σ⋅Δ**Π****	−**11.0**	−**11.0**	**σ⋅Δ**Π****	−**11.7**	−**11.7**
**Starling gradient**	**8.0**	**22.0**	**Starling gradient**	−**21.7**	−**35.7**

*Table reports the expected values for capillary, interstitial and alveolar liquid hydraulic pressure (Pcap, Pint and Pliq alv, respectively) as well as for capillary, interstitial and alveolar liquid oncotic pressure (Πcap, Πint and Πliq alv, respectively). Endothelial (σ endo) and epithelial (σ epi) protein reflection coefficients are also reported. In bold, hydraulic (ΔP) and oncotic (σΠP) pressure gradients and total Starling pressure gradient. Positive values of the Starling gradient at endothelial level indicate filtration into interstitium; negative value at epithelial level indicate alveolar reabsorption.*

Pressure values are expressed in cmH_2_O; σ is a pure number. *From Hakim et al., 1993; **from Miserocchi et al., 1993; ^#^calculated from Beck and Lai-Fook, 1983; ^##^from Parker et al., 2006. Surface tension γ = 1 dyne/cm.

Table 1 reports the expected values of hydraulic pressure for the capillary, interstitial, and alveolar fluids (Pcap, Pint, and Pliq_*alv*_, respectively) as well as those of oncotic pressure (Πcap, Πint, and Πliq_*alv*_, respectively). The value of Pliq_*alv*_, the pressure of the liquid phase coating the alveolar surface, was estimated from the equation $Pliq_{alv}=Palv-\frac{2\gamma }{R}$, (Beck and Lai-Fook, 1983). We considered Palv = 0 at end-inspiration and at end-expiration (FRC) during spontaneous breathing, and we assumed a physiological value for γ = 1 dynes/cm substantially unchanged up to 70% TLC (Bachofen et al., 1970). We assumed an alveolar radius of ∼ 50 μm at TLC (Beck and Lai-Fook, 1983) and its decrease at FRC being proportional to (FRC/TLC)^1/3^ (equal to 0.6). Accordingly, given the low value of γ, the ratio 2γ/R = Pliq_*alv*_ is ∼ 0 cmH_2_O. Clearly, the large change in trans-epithelial Starling gradient reflects the remarkable change in Pint.

Endothelial and epithelial protein reflection coefficients (σ_*endo*_ and σ_*epi*_, respectively) are also reported. Hydraulic (ΔP) and oncotic (σΠP) pressure gradients and the total Starling pressure gradient are reported in bold. Concerning σ, it was experimentally derived at high flow rates as σ = 1 −ϕ (ϕ being the lymph/plasma protein partition coefficient). However, this condition might not provide a σ value corresponding to the physiological condition of very low flow rates. For both endothelium and epithelium, we assumed σ = 0.85 (Parker et al., 2006), a higher value compared to those previously provided by Parker et al. (1978). Furthermore, under healthy conditions, the epithelium is almost totally impermeable to proteins (Gorin and Stewart, 1979), and water transport can only occur *vi*a coupled Na^+^ active absorption (Matthay et al., 1982). One report showed that epithelial monolayers generally have an electrical resistance that is an order of magnitude higher than that of endothelial monolayers and much lower solute diffusion permeability (Parker et al., 2006).

Concerning the microvascular district, one should add the important notion that functional compartmentation has been described considering a “true” alveolar compartment and an extra-alveolar capillary compartment. The latter has higher Lp and lower surface area compared to the “true” alveolar district, which has a much lower Lp but an incredibly much higher surface. Definitely, the “true” alveolar compartment only contributes about 6% of total convective albumin flux recovered in the lung lymph (Parker, 2007). Therefore, under physiological conditions, despite the existence of trans-endothelial and trans-epithelial pressure gradients shown in Table 1 (referring specifically to the “true” alveolar district), minimal fluxes occur thanks to low permeability coefficients. One shall consider that capillary surface area (A) might differ among humans, reflecting the individual extension of the alveolar capillary network that was found to vary by ∼3 fold based on the estimate of the capillary blood volume (Vc) (Miserocchi et al., 2008).

## The Response to the Increase in Microvascular Filtration

**FIGURE 4:**
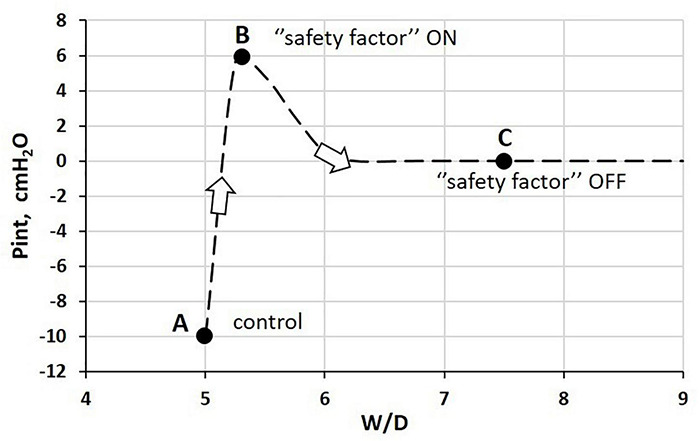
Relationship between interstitial hydraulic pressure (Pint) and wet-to-dry weight (W/D) ratio at end-expiration. Point A corresponds to physiological condition; point B to the “safety factor” (see text for explanation); point C corresponds to the absence of the “safety factor” reflecting the remarkable loss of integrity of the interstitial matrix (Miserocchi et al., 1990, 1993).

Figure 4 shows how Pint varies with increasing water balance as expressed by the W/D ratio. Point A corresponds to the physiological condition, while Point B corresponds to the physiological response acting to buffer an increase in microvascular filtration in response to an edemagenic condition.

**FIGURE 5:**
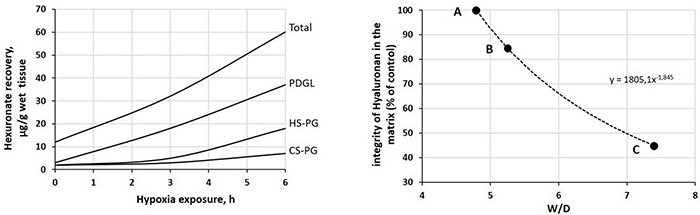
**Left:** time course of the recovery of fragments of proteoglycan families upon 12% O_2_ exposure (redrawn from Miserocchi et al., 2001). Peptidoglycans (PDGLs) control the permeability of the glycocalyx; heparin-sulfate proteoglycans (HS-PGs) control the permeability of endothelial and epithelial cells; chondroitin-sulfate proteoglycans (CS-PGs) provide rigidity to the interstitial matrix. **Right:** relationship between loss of integrity of interstitial hyaluronan (a hydrophilic non-sulfated glycosaminoglycan) and increase in W/D ratio. Points A, B, and C correspond to those reported in Figure 4.

The latter may occur because of a variety of reasons, such as hyperventilation and increase in cardiac output on sustained exercise (e.g., marathon running) (Zavorsky et al., 2014). The lung is well-equipped to respond to increased microvascular filtration due to specific morpho-functional features of the interstitial tissue structure. Indeed, increased free water filtered in the interstitium is captured by hyaluronan to form gel, whose increase in steric hindrance causes a remarkable increase in Pint from −10 cmH_2_O (Figure 5, point A) to ∼ +5 cmH_2_O (point B) (Miserocchi et al., 1993). At point B, the W/D ratio does not exceed 5.5 (10% increase relative to the control value), reflecting low tissue compliance (∼0.5 ml.mmHg^–1^⋅100 g of wet weight^–1^) (Miserocchi et al., 1993). As long as the filtration coefficient and the protein reflection coefficient remain within physiological values, interstitial gel formation provides a “safety factor” against edema formation to face an increase in microvascular filtration. Indeed, the increase in interstitial pressure (see Eq. 1) prevents further filtration and may actually favor fluid re-absorption (see Paragraph 8 for estimate of actual Starling pressure gradients). The correlation between rigidity of the extracellular matrix and low microvascular permeability was confirmed in a micro-nano biomimetic system by growing endothelial and epithelial cell layers on opposite sides of a porous mesh mimicking the extracellular structure of the basal lamina in the lung. Varying the rigidity of the mesh, and using different concentrations of polycaprolactone with gelatine, it was found that effective barrier properties were actually increased by increasing the stiffness of the matrix (Higuita-Castro et al., 2017).

Respiratory mechanics was monitored by low-frequency forced oscillation technique (FOT) in closed-chest mechanically ventilated rats under conditions referring to points A and B (Dellacà et al., 2008). The results suggested that the changes in reactance and resistance when shifting from A to B could reflect the changes in viscoelastic properties of the lung tissue, in the absence of the accumulation of extravascular fluid into the alveoli. It was, therefore, proposed that the estimate of lung mechanics with FOT might represent a non-invasive, potentially clinically useful tool to detect the earliest stages of perturbation of lung fluid balance before the condition becomes life-threatening. The next paragraph describes how the loss of safety factor (Figure 4, point C), due to fragmentation of the interstitial matrix structure, leads to a decrease in Pint to 0 cmH_2_O and to a critical increase in W/D ratio.

## The Tissue Damage and the Critical Increase in W/D Ratio

The inflammation caused by a sterile (e.g., severe hypoxia, hyperoxia, surgery, excessive parenchymal stress/strain) or bacterial/viral mechanism may trigger the fragmentation of the macromolecular architecture of the interstitial compartment, involving the whole proteoglycan family. Figure 5 (left) shows, as an example, the time course of the recovery of fragments of proteoglycan families on 12% O_2_ exposure (Miserocchi et al., 2001). Over time, fragment recovery occurs at a greater rate for PDGL and HS-PG, compared to CS-PG. The loss of integrity of the native architecture of the proteoglycan family of the interstitial matrix leads to a combination of two effects: (i) increase in microvascular permeability, which weakens the restriction to fluid leak, and (ii) increase in tissue compliance that brings Pint back to 0 cmH_2_O (Figure 4, point C, W/D ∼7.5). As a consequence, the “safety factor” is abolished and the corresponding increase in W/D ratio reflects the increase in filtration rate leading to the development of edema. The right panel of Figure 5 shows the negative correlation between the loss of integrity of the interstitial matrix and the increase in W/D. With 50% fragmentation of native hyaluronan, W/D increases by up to ∼ 7.5, a frankly edematous state (Negrini et al., 1996; Miserocchi et al., 2001).

Differences in the sequence of a fragmentation of proteoglycans may be demonstrated among various models of experimental pulmonary edema (Negrini et al., 1996; Miserocchi et al., 1999). Thus, there may be a variable time-dependent contribution to edema formation due to the increase in microvascular permeability (due to the fragmentation of HS-PG and PGDL) and/or increase in tissue compliance (reflecting the fragmentation of CS-PG). The inflammatory-dependent activation of proteases (MMP-2 and MMP-9) contributes to proteoglycan fragmentation (Passi et al., 1999). Heparan-sulfate disassembly has been reported by recent findings of HA exudates in the alveolar spaces of the lungs of patients with coronavirus disease-2019 (COVID-19) (Hellman et al., 2020).

It is noteworthy to consider the difference in timing between the phase of fragmentation of the interstitial mesh (3–6 h) and the immunity response reported for patients with COVID-19 that develops with a much slower kinetics during the first week after infection (Hou et al., 2020; Long et al., 2020).

## Causes of Lesion of the Air-Blood Barrier

### Severe Acute Respiratory Syndrome Coronavirus-2

Sepsis is due to lung lesions (Bachofen and Weibel, 1977). Concerning the kinetics of severe acute respiratory syndrome coronavirus-2 (SARS-CoV-2), a tempting hypothesis is that the entrance of virus into the ABB might resemble that of engineered nanoparticles (NPs) delivered through the airways for therapy. Findings highlight the intimate connections between viruses and NP with host lipid interactions. Both viruses and NPs could exploit dual entry, namely, crossing the plasma membrane or uptake *via* endosomes. Human ACE2-binding interface tends to have a predominantly negative electrostatic potential, allowing interaction with the SARS-CoV-2 S protein (Hassanzadeh et al., 2020). It takes less than 1 h for NPs to move into the lung interstitial space and reach the circulatory system (Dal Magro et al., 2017). Recent data suggest that heparan-sulfate proteoglycan on cell surface facilitates the attachment of SARS-CoV-2 particles to the cell surface to promote cell entry (Zhang et al., 2020). In particular, it was shown that SARS-CoV-2 spike protein interacts with both heparan-sulfate and the binding domain of ACE2 (Clausen et al., 2020). Vascular and thrombotic complications, such as symptomatic acute pulmonary embolism, deep-vein thrombosis, ischaemic stroke, myocardial infarction, and systemic arterial embolism, have been reported in severe COVID-19 cases (Farhangrazi et al., 2020).

### Reactive Oxygen Species

Molecular oxygen is activated in either hyperoxia or hypoxia into reactive oxygen species (ROS). Effective antioxidant defenses counteract the reactivity of ROS. However, the overwhelming production of ROS coupled with their insufficient scavenging by endogenous antioxidants will lead to tissue damage and cell death (Kulkarni et al., 2007). ROS are implicated in the increase in alveolar permeability (Waxman and Kolliputi, 2009; Kolliputi et al., 2010). It was found that exposure to 100% O_2_ increased alveolar epithelium permeability similar to that caused by the alloxan model of lung edema, and that this occurred before the onset of interstitial or alveolar edema (Matalon and Egan, 1981, 1984).

Hyperoxia also causes damage to endothelial cells (Kistler et al., 1967) and tissue structure (Chow et al., 2003; Kallet and Matthay, 2013) because of increased production of reactive oxygen species (ROS) at mitochondrial level (Freeman and Crapo, 1981).

The risk of ROS-dependent lung injury is reported to occur at FIO_2_ > 0.7, and may worsen at FIO_2_ > 0.8 for prolonged exposure. To limit the exposure to high levels of FIO_2_, there is a recommendation by ARDS Network to target PaO_2_ levels between 55 and 80 mmHg in mechanically ventilated patients. Milder levels of hyperoxemia would be supported by data suggesting that PaO_2_ > 80 mmHg is associated with worse clinical outcomes at all levels of acute respiratory distress syndrome severity (Aggarwal et al., 2018).

Type-1 epithelial (Epi1) cells could be completely destroyed by hyperoxia (Weibel, 1971), so, after a few days of hyperoxia exposure, the whole alveolar surface would be found to be lined only by cuboidal Epi2 cells (Kapanci et al., 1969; Kaplan et al., 1969), a finding also reported for oxygen poisoning in human lung (Nash et al., 1967). The role of Epi2 cells was interpreted as that of regeneration of new Epi1 cells (Adamson and Bowden, 1974; Bachofen and Weibel, 1974; Evans et al., 1975). Neo-formed Epi1 cells appear able to branch and form at least four or even more lining units on endothelial cells (so called “epithelial plate”) (Weibel, 2015). Ischemia-reperfusion injury (a primary graft dysfunction) is a form of high-permeability pulmonary edema due to endothelial lesion (Liang et al., 2019).

### Lung Overdistension

Lung overdistension is a major cause of tissue lesion. Going from low to high transpulmonary pressure causes an increase in fluid filtration rate (Bo et al., 1977). Inflating the lungs to total capacity is found to cause free solute movement across the lung epithelium because of larger pore radii. Furthermore, decreasing lung volume does not produce a smaller pore radius that either remains the same or becomes larger. This phenomenon is interpreted as depending on permanent lung lesion (Egan, 1980). Furthermore, data by Egan (1982) suggest that acute lung distension at Palv = 40 may cause epithelial albumin leak but not alveolar flooding: the interpretation of this result is that, upon short exposure to considerable lung distension, no time is given for remarkable matrix fragmentation. A 5-fold increase in Jv over 6 h with step change of Palv from 10 to 20 cmH_2_O (Tarbell et al., 1999; Tarbell, 2010). W/D was found to increase from 6.63 to 7.45 over 2 h with Palv up at 48 cmH_2_O (Yoshikawa et al., 2004); this same study reported an increase in albumin concentration in bronchoalveolar lavage fluid by up to 250 mg/ml.

Other data report a significant increase in endothelial Kf for alveolar pressure exceeding 42 cmH_2_O (Parker et al., 1984). A remarkable increase in Lp was found over 8h due to increased size of pores, and thus increase in filtration surface area, for only 10% increase in strain (lung-on-a-chip microdevice model, Huh et al., 2012). Experimental models of an injurious ventilatory strategy were found to predispose to subsequent bacteremia and associated impaired host defense (Lin et al., 2003).

### The Alveolar Folding/Unfolding Zone

The specific topology of Epi1 cells is such that they are far from being flat (see Figure 2; Weibel, 2015). Furthermore, these cells present numerous pleats on their surface, as deduced by following the profile of the basement membrane, that are subjected to the cyclic folding/unfolding process on changing lung volume (Bachofen et al., 1987). Based on data from electron microscopy, unfolding was found to be completed in healthy lung upon reaching a transpulmonary pressure ∼ 15 cmH_2_O, corresponding to ∼75–80% Vital Capacity (VC), (with a surface tension not exceeding 5–7 dynes/cm, Bachofen et al., 1987). The folding-unfolding process was confirmed also for the human lung by estimating the increase in lung diffusion capacity on increasing lung volumes from FRC up to 100% VC. It was found that the increase in diffusion capacity could be modeled according to the increase of the ratio between the alveolar surface and thickness of the air-blood barrier (Salv/τ) (Miserocchi et al., 2008).

**FIGURE 6:**
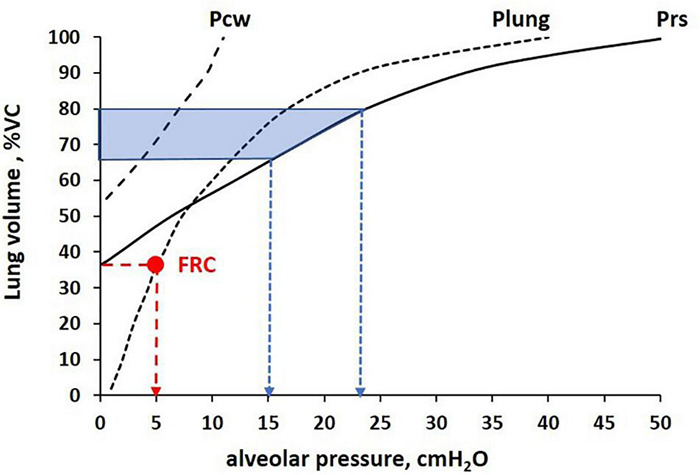
Volume-pressure curve of the lung, chest wall, and total respiratory system (Plung, Pcw, Prs, respectively) to identify the relative lung distention at functional residual capacity (FRC) (Palv = 5 cmH_2_O, red arrow) and completion of the alveolar unfolding process occurring in the range Palv 15–25 cmH_2_O (blue arrows), corresponding to ∼65–80% Vital Capacity (VC, shaded area).

The result was that the increase in diffusion capacity was proportional to the increase of Salv up to a lung volume of ∼80% VC, at which the “reserve” surface of the unfolding pleats was completed. Above this volume, the diffusion capacity was proportional to the decrease in τ. It was, therefore, concluded that above ∼80% VC, thinning of septa occurred because of direct tissue stretching. Further data confirm that in the healthy lung, increasing volume might occur without much strain of the Epi1 cell surface thanks to the “reserve” surface of the pleats, and that direct stretching of the septa occurs with increasing strain above 75–80% VC (Knudsen and Ochs, 2018). Figure 6 shows the volume-pressure curve of the lung, chest wall, and respiratory system in a healthy subject to show: (i) the FRC (red dot) and (ii) the percent of lung distention (65–80% of VC) and corresponding alveolar pressure (15–25 cmH_2_O) upon approaching the saturation of the alveolar unfolding process (shaded area).

In the supine subject, FRC is decreased down to about 22% (Agostoni and Mead, 1964) so, assuming that the saturation of the unfolding process also occurs at about 80% of lung distension, the range of the folding/unfolding process is increased.

One may attempt to comment on the clinical outcome from mechanically ventilated patients with ARDS considering the ventilatory strategy with specific relation to the folding/unfolding zone. One can, therefore, pose the question of how a ventilatory strategy implying a cycling involvement of the folding/unfolding zone might have an impact on lung function. Protti et al. (2013) reported 50% increase of death when oscillating lung volume across 70% lung distension, compared to a strategy of ventilation set either above or kept below 70% VC. The occurrence of repetitive recruitment and de-recruitment during mechanical ventilation of a diseased lung (bleomycin treatment or surfactant deactivation by Tween 20 detergent) was shown to cause remarkable alveolar instability with atelectasis in some alveoli and overdistention of adjacent ones, referred to as atelectrauma and volutrauma, respectively (Perlman et al., 2011; Rausch et al., 2011; Cressoni et al., 2014; Makiyama et al., 2014; Bates and Smith, 2018; Knudsen and Ochs, 2018; Retamal et al., 2018; Bates et al., 2020; Gaver et al., 2020). Accordingly, it was hypothesized that these maneuvers set a vicious cycle by which the alveolar damage progresses within the lung from atelectatic to adjacent non-atelectatic lung regions through mechanical interdependence. Data from Hamlington et al. (2018) also prove that the sequence of cyclic folding/unfolding leads to a remarkable increase in microvascular permeability due to progressive increase in endothelial lesions (rich-get-richer scheme). It appears, therefore, that a ventilatory strategy implying a cyclic folding/unfolding process favors tissue damage.

The Rest Lung Approach (RLA) (Acute Respiratory Distress Syndrome Network et al., 2000) and Open Lung Approach (OLA) (Writing Group for the Alveolar Recruitment for Acute Respiratory Distress Syndrome Trial (ART) Investigators, 2017) are the two main protective ventilation strategies being used currently. The RLA assumes the two-compartment ARDS model with collapsed, edematous, and unstable tissue in the dependent portion of the lung and small volume of normal tissue in the non-dependent portion of the lung (Gattinoni and Pesenti, 2005). Using a low tidal volume and plateau pressure, the RLA is designed to “Rest” the dependent injured lung tissue by keeping it completely out of the ventilation cycle and “Protect” the small amount of normal lung tissue from overdistension. The OLA assumes that lung protection is better afforded by completely opening the collapsed and edema-filled tissue using higher PEEP with and without recruitment maneuvers. Unfortunately, these protective ventilation strategies have not been effective in further lowering mortality over the last 20 years, suggesting the need for novel protective ventilation strategies to be tested (Caser et al., 2014; Fan et al., 2018).

Airway pressure release ventilation (APRV), set and adjusted using the Time-Controlled Adaptive Ventilation (TCAV™) method^1^, sets ventilator pressure above the folding/unfolding zone and uses a very brief release phase, personalized to changes in lung collapse time constants, that does not give the tissue sufficient time to fold. The TCAV™ method has been reported to be protective as compared to cyclic ventilation crossing the folding/unfolding zone (Roy et al., 2012, 2013a,b; Kollisch-Singule et al., 2014a,b, 2015). A reduced microstrain has been reported for a ventilation implying alveolar pressure above the folding/unfolding zone (PEEP 16–24 cmH_2_O) and a short time at low pressure (Kollisch-Singule et al., 2014a) using the TCAV™ method. Data from Yoshida et al. (2009) indicated that APRV with Palv 21 cmH_2_O was more efficient than Pressure Support Ventilation (PSV) set at PEEP 10 with Ppeak 23 cmH_2_O that implies cycling crossing of the folding/unfolding zone. In a bleomycin injury lung model, tissue damage could be moderated by ventilation at PEEP = 20 cmH_2_O, where unfolding is almost completed (Knudsen and Ochs, 2018). Finally, a multilevel analysis of data from 3,562 patients with ARDS revealed that the least risk of death in the hospital correlated with a ventilatory strategy at alveolar pressure high enough to avoid a wide range of cycling crossing of the folding/unfolding zone (Amato et al., 2015). It has also been shown that in preterm infants (≤32 weeks of gestation), alveolar recruitment is more efficient with continuous than with discontinuous CPAP (∼5 cmH_2_O), which normalizes the FRC volume (Lam et al., 2020).

The TCAV™ method, which is a novel Stabilizing the Lung Approach (SLA), was developed based on the fact that cyclic recruitment-derecruitment in surfactant-deprived lungs leads to a significant degree of alveolar instability (Nieman et al., 2020b). The goals of the SLA are to very quickly stabilize alveoli and prevent folding/unfolding injury. Once stable, the collapsed tissue can be gradually reopened over an extended period of time (hours or days). Using a very brief release phase, directed by changes in lung collapse time constants, has been shown to very quickly stabilize alveoli. The extended inspiratory time, known as the continuous positive airway pressure (CPAP) phase, gradually opens the collapsed lung tissue over an extended period of time (Roy et al., 2012, 2013a,b; Kollisch-Singule et al., 2014a,^2015^). The SLA may facilitate edema re-adsorption and reduce mechanical damage to endothelial and epithelial cells, as compared with the RLA and RLA methods (Nieman et al., 2020a,b). The possible mechanisms for the efficacy the TCAV™s methods in reducing edema accumulation and expediting edema removal are based on the following: (i) very quickly avoiding the cycling folding/unfolding of alveolar walls using a very brief release phase, (ii) the rapid lung inspiration at the end of the release phase gradually recruits alveoli *via* a “ratchet” mechanism similar to the way the newborn opens their collapsed fluid-filled lungs at birth (Tingay et al., 2021), (iii) stabilizing and then progressively opening alveoli will maintain normal surfactant protein concentration.

### Patient-Self-Inflicted Lung Injury

The case of the patient-self-inflicted lung injury (*P-SILI*) deserves a particular consideration. Evidence has been provided for high tidal volumes developed by patients receiving non-invasive respiratory support based on increasing alveolar pressure. Furthermore, a correlation was found between diaphragmatic swings and risk for P-SILI due to lung overdistension (Carteaux et al., 2016; Brochard et al., 2017). The correlation between lung overdistension and lung lesion can be easily accepted (Cruces et al., 2020). However, the understanding of potential “exaggerated” diaphragm contractions has remained elusive. Perturbation in respiratory drive has been invoked by reviewing some possible reflex mechanisms of neural control of breathing (Brochard et al., 2017; Telias et al., 2018). We wish to consider a so far overlooked physiological feature of the diaphragm as a pressure generator. For a given neural drive, diaphragmatic contraction appears to be quite stable and cannot be modified once started, as the diaphragm is almost void of proprioceptors (Corda et al., 1965). From a mechanical standpoint, the question is: what is the outcome of diaphragmatic contraction when the diaphragm is facing a change in mechanical load? If the load is increased (e.g., adding external respiratory elastance), the inspired volume will decrease (Pengelly et al., 1971). In fact, no “load compensating reflex” is present, as no proprioceptor afferent input is present. The opposite case is now that of removing part of the elastance that the diaphragm faces during its contraction. This case occurs by increasing alveolar pressure; in fact, increasing alveolar pressure might remove part or all of lung elastance. The result is an increase in lung volume, since the first breath gives rise to over distension and lung injury (Pengelly et al., 1971). One could estimate that by removing the elastance roughly equivalent to that of the lung, the increase in inspiratory volume would amount to ∼150–200% of the control value. Such increase is expected to vary among patients, as lung elastance is related with the number of ventilated units, being higher with increasing alveolar flooding. Neuromuscular blockers obviously contrast this phenomenon (Forel et al., 2006; Papazian et al., 2010). External (inspiratory) intercostal muscles do exhibit proprioceptors innervation: accordingly, they might increase their contraction on adding external elastance (“load compensating reflex”) but would be silent on increasing alveolar pressure (thus, the decrease in elastance remains unopposed). One could consider that a patient exposed to increased alveolar pressure has to perform active expiration that is normally passively relying on the elastic recoil of the respiratory system. If the above interpretation is valid, it would not be paradoxical to retain the P-SILI acronym, but the expansion should read Patient-Strategy Induced Lung Injury!

## Factors Preventing the Development of Edema: Interstitial Capacitance, Lymphatic Flow, and Vasomotion

The first line of defense against increased microvascular filtration is fluid accumulation in the thick portion of the air blood barrier. Up to W/D ∼5.5, some fluid accumulates in the endothelial cells whose swelling may account for ∼45% of their control volume, while ∼85% of filtered fluid accumulates in the thick portion of the air-blood barrier (Conforti et al., 2002) where lymphatics are present. This morphological arrangement corresponds to a model of a relatively rigid initial compartment (the thin portion of the ABB) that communicates by a relatively high-resistance pathway to a larger downstream capacity (the thick portion of the ABB) (Unruh et al., 1984).

Human lymphatics extend deep inside the pulmonary lobule; indeed, a peripheral distribution of lymphatics (defined as interstitial lymphatics) was described down to the peri-microvascular district near the alveolar, the alveolar ducts, and the interalveolar septa (Schraufnagel, 1992; Hainis et al., 1994; Schraufnagel et al., 1994; Weber et al., 2018). These lymphatics converge into a central compartment, becoming large conducting vessels associated with the broncho-vascular bundle (Weber et al., 2018).

Under baseline conditions, lymph flow rate is 0.063 ml/min/100 g (Effros and Parker, 2009), and only about 4% of this value corresponds to alveolar fluid clearance. The latter corresponds to the transepithelial active sodium transport. If the “safety factor” is intact, most fluid clearance occurs *via* Starling-depended fluid re-absorption (Miserocchi, 2009) back into the capillaries, with only a minor fraction (∼18%) *via* lymphatics (Pearse et al., 1993).

Lymphatics can increase flow, and thus, provide a passive negative-feedback control loop to offset an increase in extravascular volume (Miserocchi, 2009). Interestingly, lymph flow was found to be proportional to the rate of increase in lung weight that is directly related to the microvascular filtration rate (Mitzner and Sylvester, 1986). Interstitial fluid ought to percolate through the interstitium down a pressure gradient generated by lymphatics themselves that were shown to generate a subatmospheric pressure compatible with the measured value of Pint (Miserocchi et al., 1989). Redistribution of fluid downstream from filtration sites was modeled by relating the increase in lymphatic flow in response to increase in filtration rate as a function of interstitial fluid resistance (Roselli et al., 1984). These authors found that, for interstitial compliance in the physiological range (intact matrix), a 3-fold increase in lymphatic flow could balance the increased filtration rate by holding interstitial pressure at about 2 cmH_2_O. When interstitial hydration increases, matrix fragmentation would actually decrease tissue resistance for fluid flux toward lymphatics, in line with the model developed by Unruh et al. (1984). In case of experimental lung lesion, lung lymph flow was found to increase by ∼8- to 10-fold (Rutili et al., 1982).

A recent microfluidic study revealed considerable differences among lung regions concerning interstitial fluid dynamics from capillary filtration sites, through the porosity of the interstitium, and into the lymphatic network. This conclusion came about by combining specific lymphatic immunohistochemistry with high-resolution X-ray computed tomography and finite-element mathematical modeling that identified differences related to the functional interaction between tissue mechanical properties and the action of the lymphatic pump (Robinson et al., 2019). Lymphatic clearance of alveolar fluid can only occur with an epithelial barrier lesion, as confirmed by data from hyperoxia (Hainis et al., 1994; Schraufnagel et al., 1994) and ventilator-induced lung injury lesion models (Schraufnagel et al., 2003).

Mechanical ventilation and positive end-expiratory pressure (PEEP) impede lung lymph flow by increased intrathoracic pressure and increased central venous pressure. PEEP may, thus, enhance edema formation of the lung (Hedenstierna and Lattuada, 2008). Obviously, when lung W/D increases, the lymphatic flow cannot balance the flow of filtration. This occurs in case of severe endothelial and epithelial lesions. Alveolar flooding occurs when the increase in lung W/D exceeds 35% of baseline (Taylor and Parker, 1985), corresponding to ∼6.25.

**FIGURE 7:**
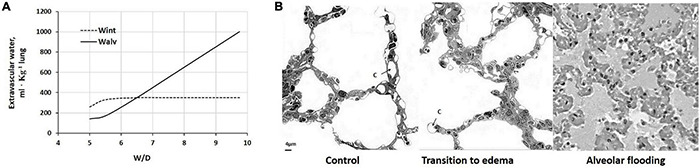
**(A)** Distribution of extravascular lung water to the interstitial (Wint) and alveolar (Walv) compartment on increasing W/D. **(B)** Morphological changes of the lung from control (left) to a transition phase with fluid accumulation in the interstitial space (center) and alveolar flooding (right). c, pulmonary capillary (redrawn from Miserocchi, 2007).

Figure 7A shows the distribution of extravascular interstitial and alveolar water upon increasing W/D by up to 10, normalized to 1 kg lung (Cressoni et al., 2013) and assuming ∼35% increase in interstitial water (Negrini et al., 2001b). Figure 7B provides images of the morphological changes of the lung from control (left) to a transition phase implying fluid accumulation in the interstitial space (center) and alveolar flooding (right). On mechanical ground, alveolar flooding implies a progressive loss of alveolar expandable units, and correspondingly, a decrease in lung compliance.

Based on the pressure generating a trans-endothelial flow, a morphological-based model was developed to describe the interaction between control of extravascular lung water and capillary perfusion under edemagenic conditions (Mazzuca et al., 2016). The model focused in particular on the fact that the increase in Pint, besides buffering and even reversing the transcapillary Starling gradient, leads to capillary de-recruitment because of the compressive effect of positive interstitial pressure; the resultant decrease in exchange surface area is a potent factor to decrease Kf. Data on humans confirmed blood flow limitation in edemagenic lung regions, despite the administration of a vasodilator agent (Scherrer et al., 1996).

Precapillary vasoconstriction is a further strong anti-edemagenic mechanism (Negrini et al., 2001a; Mazzuca et al., 2019). In fact, redirection of blood flow from edematous to normal lung regions has been correlated with the increase in Pint in the former as well as with the corresponding precapillary vasoconstriction and vasodilation in the two regions, respectively (Rivolta et al., 2011). Interestingly, inter-individual differences have been documented concerning capillary recruitment/de-recruitment under edemagenic conditions, such as exercise or exposure to hypoxia; in particular, capillary de-recruitment has been interpreted as individual proneness to develop lung edema (Bartesaghi et al., 2014; Beretta et al., 2017a,b). In general, a greater density of pulmonary capillaries favors oxygen diffusion-transport at the alveolar level (Beretta et al., 2017a,b); however, it involves a greater risk factor of developing lung edema because of greater capillary surface for microvascular filtration (Mazzuca et al., 2016). It is tempting to consider pulmonary hypertension, as observed under strong edemagenic conditions (hypoxia), as the consequence of precapillary vasoconstriction represents a powerful means to limit microvascular filtration (Negrini et al., 2001a; Mazzuca et al., 2019).

There is an interesting report concerning the preventive effect of gadolinium in models of lung lesions caused by Palv at ∼30 cmH_2_O that induces a ∼2-fold increase in Kf. The data suggest that gadolinium prevents increased microvascular permeability caused by stretch-activated cation channel-induced increases in intracellular calcium concentration (Parker et al., 1998). Another potentially edema-preventive strategy considers the role of NADPH oxidase type 2 (NOX2) that is a major source of ROS in the lung. Pre-treatment with an inhibitor of NOX2 (Fisher et al., 2021) was hypothesized as preventive of the secondary inflammatory component. A further study suggests that dietary antioxidants could represent a potential treatment for oxidative stress lung injury in patients on mechanical ventilation (Patel et al., 2020).

## Modeling the Starling Gradients in the Progression Toward Severe Lung Edema

Table 2 summarizes the database to compare the Starling gradients as edema progresses from point B (“safety factor”) to point C. When the “safety factor” is on, trans-endothelial Starling gradients are decreased; in particular some fluid reabsorption may actually occur across the capillary wall at end-expiration. This last point is confirmed by Pearse et al. (1993) who, adopting an experimental model that limited the increase of W/D at ∼6, found that ∼42% of water filtered into the interstitium was cleared by reabsorption into the pulmonary capillaries. At point B, considering γ is still equal to 1 dynes/cm, negative values of Starling gradients across the epithelium prevent any risk of alveolar flooding.

**TABLE 2:** Trans-endothelial and trans-epithelial Starling pressure gradients at end-expiration, with “safety factor” (point B in Figure 4) and after some degree of matrix fragmentation (point C in Figure 4).

	Trans-endothelial			Trans-epithelial	
	Point B (W/D = 5.5)	Point C (W/D∼7.5)		Point B (W/D = 5.5)	Point C (W/D∼7.5)
Pcap*	9	9	Pint**	5.7	0
Pint**	5.7	0	Pliq alv^#^	∼0	−17
σ end^##^	0.85	0.5	σ epi^##^	0.85	0.5
Πcap**	26.8	14.7	Πint**	13.8	9.3
Πint**	13.8	9.3	Πliq alv	0	5
			γ	1	25
**ΔP**	**4**	**9**	**ΔP**	**5.7**	**17**
**σΔ**Π****	−**11.0**	**0.0**	**σΔ**Π****	−**11.7**	−**2.15**
**Starling gradient**	−**7.0**	**6.3**	**Starling gradient**	−**6.0**	**14.85**

*This table reports the expected values for capillary, interstitial, and alveolar liquid hydraulic pressures (Pcap, Pint, and Pliq alv, respectively) as well as for capillary, interstitial, and alveolar liquid oncotic pressures (Πcap, Πint, and Πliq alv, respectively). Endothelial (σ endo) and epithelial (σ epi) protein reflection coefficients are also reported. In bold, hydraulic (ΔP) and oncotic (σΠP) pressure gradients and total Starling pressure gradient. Positive values of the Starling gradient at endothelial level indicate filtration into interstitium; negative values at epithelial level indicate alveolar reabsorption. From B to C (the phase corresponding to progressive fragmentation of the matrix), Pint returned to zero, suggesting loss of the physiological alveolar mechanical tethering interaction (Mead et al., 1970). Accordingly, from point C on, we considered Pint = Palv, as suggested by Glucksberg and Bhattacharya (1991).*

Pressure values are expressed in cmH_2_O; σ is a pure number. *From Hakim et al., 1993; **from Miserocchi et al., 1993; ^#^calculated from Beck and Lai-Fook, 1983; ^##^from Parker et al., 2006. Surface tension (γ), dyne/cm.

We ignore the value of surface tension at point C (W/D ∼7.5), as well as the corresponding value of Pliq_*alv*_. Assuming that γ value increased by up to 25 dyne/cm at point C (due to both surfactant dilution and de-activation), Pliq_*alv*_ would decrease to −17 cmH_2_O, so the resultant Starling trans-epithelial pressure gradient would potentially cause alveolar flooding. Modeling the increase in γ between 20 and 40 dynes/cm would only slightly change the result confirming that a W/D in the range 6.5–7 represents the critical threshold for alveolar flooding.

**FIGURE 8:**
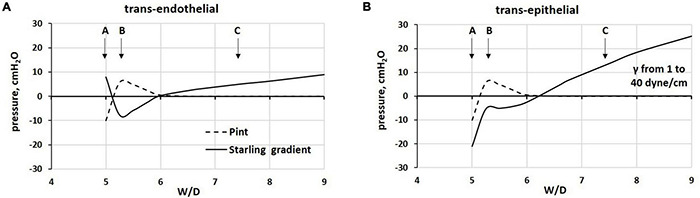
Modeling of **(A)** trans-endothelial and **(B)** trans-epithelial Starling pressure gradients (solid line) on increasing W/D, based on experimentally measured values of interstitial pressure (Pint, dashed line), with Palv = 0 cmH_2_O. Points A, B, and C as from Figure 4.

In Figure 8, we modeled the (A) trans-endothelial and (B) trans-epithelial Starling gradients on increasing W/D, in relation to the corresponding values of interstitial pressure (Pint), with Palv = 0 cmH_2_O.

With the “safety factor” on (point B), both trans-endothelial and trans-epithelial Starling gradients promote fluid reabsorption, in line with data from Egan et al. (1976, 1977). The critical factor leading to alveolar flooding is the development of a remarkably increasing negativity of Pliq_*alv*_ as a consequence of the progressive increase in surface tension (γ) due to surfactant de-activation. We assumed an increase in γ from 1 to 25 dynes/cm for W/D > 6.5 (see Table 2).

Changes in Starling gradients and permeability parameters may be combined differently within the whole lung. In fact, the available experimental evidence confirms the heterogeneity in both regional lung fluid accumulation and perfusion under edemagenic conditions (Bachofen et al., 1993). Basal lung regions are, in general, more exposed to edema because of greater blood perfusion, implying higher capillary Kf (Rivolta et al., 2011). Alveolar flooding was found to occur in a quite non-homogeneous fashion revealing considerable regional differences (Wu et al., 1995).

**FIGURE 9:**
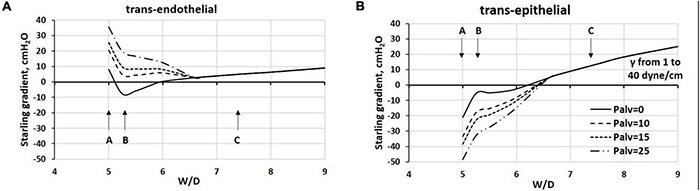
Estimated Starling pressure gradients at **(A)** endothelial and **(B)** epithelial levels on increasing alveolar pressure by up to 25 cmH_2_O, and on varying W/D from 5 to 9. Points A, B, and C refer to those in Figure 4.

Figure 9 reports the estimated Starling pressure gradients at the (A) endothelial and (B) epithelial levels with increasing alveolar pressure of up to 25 cmH_2_O, as a function of W/D. We modeled the Starling pressure gradients on increasing W/D by increasing surface tension values from 1 to 40 dynes/cm. For W/D in the physiological range (5–6.5), the increase in Palv shifts the trans-endothelial gradients toward filtration, and on the opposite, the trans-epithelial toward alveolar re-absorption. Both effects tend to wane as W/D increases. Thus, in the W/D range of 5–6.5, the increase in Palv causes capillary filtration and alveolar clearance; accordingly, lymphatics have to cope with both flows. For W/D > 6.5, both trans-endothelial and trans-epithelial gradients favor filtration, leading to alveolar flooding. Note that for W/D > 6.5, the Starling pressure gradients, referring to different Palv, overlap, reflecting the fact that when the proteoglycan matrix is disrupted, the increase in Palv impacts equally on the increase in capillary pressure, interstitial pressure (Glucksberg and Bhattacharya, 1991), and Pliq_*alv*_ (Beck and Lai-Fook, 1983).

The graphs in Figure 9 may be considered either as representing the Starling pressure-dependent course of developing edema, and as representing the regional differences in water balance within the lung. Indeed, novel CT imaging methods (Cereda et al., 2016, 2017) showed that aerated and non-aerated lung regions were diffusely intermingled. The erratic distribution of edema can be justified by the heterogeneous susceptibility to edema formation at alveolar level that was attributed to local differences in microvascular permeability possibly dependent upon alveolar morphology, as suggested by a novel lung imaging technique under experimental edemagenic conditions (Mazzuca et al., 2019). Heterogeneity in alveolar mechanics and fluid dynamics is confirmed by data from Smith et al. (2020) who generated VILI in mice ventilated with 50 cycles/min from FRC up to a plateau pressure of 37.5 cmH_2_O. Studying the alveolar de-recruitability on decreasing Palv, three alveolar phenotypes were described as a consequence of VILI: (1) flooded alveoli that cannot be recruited at any pressure, (2) unstable alveoli that are open at high pressures but readily collapse as pressure is reduced, and (3) relatively normal alveoli that remain open at low pressures.

An important relationship exists during cyclic mechanical ventilation between peak airway pressure (Ppeak) and positive end-expiratory pressure (PEEP). The data in Figure 9 suggest that the trans-endothelial filtration gradient increases with increasing Ppeak and PEEP. Conversely, no risk of alveolar flooding occurs for W/D < ∼6.5, as trans-epithelial gradients are in favor of fluid absorption. This may be partially balanced by a decrease in filtration surface as a consequence of a decrease in overall capillary blood volume (Miserocchi et al., 2008). Blood flow is also temporarily reduced, being proportional to the decrease in the difference between pulmonary artery and alveolar pressure. Lymphatic drainage can increase in this situation to maintain lung fluid balance. However, the time evolution of this situation should be regarded in terms of increase in W/D ratio as related to the progression of the fragmentation of the interstitial matrix (Figure 5).

The TCAV™ method, if applied pre-emptively before the development of severe lung injury, has been shown to prevent collapse of the acutely injury lung, maintaining a fully open lung, and if applied following the development of severe lung injury, will rapidly stabilize alveoli even with fast collapse time constants. If the ventilation strategy can put a “cast” within the acutely injured lung, much like putting a cast on a broken arm, the mechanisms of VILI-induced edema generation would be eliminated (Nieman et al., 2018).

One shall note that edema clearance in experimental models of ARDS can only occur in lung regions where W/D ratio does not exceed 6.5–7, as only in these regions the Starling gradients allow fluid reabsorption (see Figure 9). The presence of these regions can justify the observed average decrease in W/D ratio for the whole lung from about from ∼9 to ∼7; the latter value still corresponds to severe edema (Habashi, 2005; Matsuzawa et al., 2010; Roy et al., 2013a,b; Kollisch-Singule et al., 2014a,b, 2015).

## Time Constant of Developing Severe Alveolar Edema

The development of lung edema depends upon various combination of an increase in Lp and in A as well as a decrease in σ, involving both the endothelium and the epithelium. Increase in capillary pressure and lung overdistension were shown to cause damage to the endothelial and epithelial barriers (Wu et al., 1995). The critical phase of edema pivots around a W/D of ∼ 6–6.25. We hypothesize that for W/D to exceed 6, capillary filtration should occur down this still low-pressure gradient *via* increase in Lp and decrease in σ acting over a progressively larger portion of the alveolar compartment surface area, which may cause matrix fragmentation. Consider that the Lp of the “true” alveolar compartment is physiologically 20 times lower than that of the extra-alveolar compartment (Parker et al., 2006) while the capillary surface area is in the order of 70 m^2^ on a morphological basis. We suggest that a W/D ∼ 6.5 is the likely threshold, above which fluid will seep from capillary to alveoli down a small driving pressure gradient acting over a large surface of damaged air-blood barrier.

An exponential increase in lung weight (above a W/D of ∼ 6) has been modeled with a relatively short time constant (∼4 min) by moderate increase in either Lp or decrease in σ (Mazzuca et al., 2016). Similar time constants were found for the exponential increase in lung weight in response to a step increase in venous pressure (Parker and Townsley, 2004). The above hypothesis is strengthened by the finding that a >3-fold increase in microvascular filtration already causes an increase in trans-capillary convective transport of proteins compared to either diffusive or vesicular transport, suggesting lowering of the σ value (Egan et al., 1977). Other data predict that a 50% increase in lung water may occur within 45 min after a step increase in left atrial pressure (2–14 cmH_2_O) and pulmonary artery pressure (13–22 cmH_2_O) (Rutili et al., 1982).

## The Way to Recovery

A good model to explain the clearance of alveolar edema is the one proposed in full-term newborns (Miserocchi et al., 1994). Alveolar fluid clearance occurs down a two-step coupled mechanism: (1) epithelial water absorption *via* Na^+^-dependent transport and (2) Starling-dependent capillary re-absorption. Note that fluid reabsorption into lung capillaries is a self-limited mechanism due to increase in the colloid osmotic pressure at the interstitial end of the glycocalyx generated by protein accumulation preventing further absorption (Curry, 2005; Levick and Michel, 2010; Mazzuca et al., 2019). A recent report (Tingay et al., 2021) shows that the transition from fluid-filled to aerated lungs in full-term newborns is accomplished using ventilation patterns characterized by a rapid high-peak inspiratory flow to “ratchet” open collapsed and flooded airway. The collapse of these newly recruited airways during expiration was minimized by partially closing the glottis to keep the lung pressurized. Thus, the inspiratory phase allows for rapid lung aeration extending from the central to distal lung portions; the expiratory phase maintains alveolar pressure allowing for fluid clearance. Maintaining a pressurized lung during expiration in a lung with low permeability of the epithelium and low compliance of the interstitial matrix results in an increase in interstitial pressure (as at point B) generating a trans-endothelial absorption *via* the Starling gradient. These two features are incompletely developed in preterm lung, and therefore, hinder alveolar fluid reabsorption (Miserocchi et al., 1995). An intact epithelial barrier allows Na^+^-dependent alveolar fluid reabsorption. Matthay and Wiener-Kronish (1990) provided the first evidence in humans to support the hypothesis that active ion transport across the alveolar epithelial barrier is the primary mechanism for the clearance of edema fluid. Na^+^ transport across the distal airway epithelium is the main determinant of alveolar fluid clearance, as demonstrated in several different species, such as the human lung (Berthiaume and Matthay, 2007). With an intact epithelium, the clearance of alveolar fluid is rapid in patients with severe hydrostatic edema (Verghese et al., 1999). In contrast, in the majority of patients with acute lung injury, alveolar fluid clearance is impaired by reduced Na^+^ absorption (Ware and Matthay, 2001).

Controversies regarding the optimal mechanical ventilation strategy in patients with severe edema centers around the maintenance of alveolar pressure that favors fluid clearance and avoids the complication of volutrauma and atelectrauma. To favor alveolar fluid re-absorption in experimental edema models, the ventilatory strategy should allow to decrease W/D below 6.5, a cut-off suggesting restoration of control of lung fluid balance (Figures 8, 9).

The re-deposition of an interstitial macromolecular matrix assuring low microvascular permeability and low interstitial tissue compliance appears critical for the control of lung fluid balance, since this would re-establish a “safety factor.” Most endothelial but not epithelial cells appear to be involved in the mechano-transduction signaling process in response to edemagenic conditions (Botto et al., 2006) that influence a specific cascade of cellular events aiming at remodeling of matrix macromolecules (Palestini et al., 2002, 2011; Daffara et al., 2004). There are indications that post-infection activation of fibroblast does not allow the re-establishment of a matrix composition similar to the native composition (Li et al., 2016). In particular, the activation of fibroblast impacts hyaluronan synthase that controls the deposition of bundles of hyaluronan that actually represents the fibrotic response to recovery. In the presence of hyaluronan fragments during the early phase of lung injury, the balance between hyaluronan synthase isoforms and hyaluronidases plays an important role in the pathogenesis of lung fibrosis (Li et al., 2000). Recent data suggest that hyaluronan synthesis occurs in a matter of days (Bell et al., 2019).

## Conclusion

Although the sequence of events leading to severe lung edema have been delineated, at this point, there remains a wide gap in our knowledge of how to prevent the loss of lung fluid balance when W/D > 6 and the critical role of properly set mechanical ventilation. The data from this analysis support the contention that in diseased portions of the lungs (W/D > >6.5) the mechanisms necessary to clear edema fluid have been lost. Since the distribution of edema is heterogeneous by nature, in the lung portions where W/D < 6.5, the control of lung fluid balance is only possible when the built-in safety factors, intact matrix, interstitial capacity, and lymphatics not yet saturated, are still functional. Once both barriers (endothelium and epithelium) are breeched, and built-in safety components (capacity reservoirs and lymph flow) are saturated, massive flooding occurs. These considerations may help to “debug” the controversies concerning the outcome of various experimental models of lung lesion or clinical outcome in patients. Comparing data from different ventilatory strategies appears difficult, as these are highly influenced by the number of alveolar units allowing fluid clearance that cannot be directly measured. Reported values for W/D of the whole lung to validate ventilatory strategies aimed at facilitating edema re-adsorption, reducing mechanical damage, and preventing further increase in permeability never fell below 6.8 (see Paragraph 8). So far, neither CT scan nor ultrasound has been correlated to the corresponding regional values of W/D ratios. It would be useful to identify a non-invasive tool to detect the earliest stages of perturbation in lung fluid balance before the condition becomes life-threatening. Ventilatory support strategies, during either spontaneous breathing or mechanical ventilation, should carefully balance factors that can harm functioning alveolar units. Aiming at high oxygen saturation at the expense of alveolar hyperoxia should also be considered (see Paragraph 6.2). The clinical problem appears to be that of avoiding damage to the alveolar units that still retain gas diffusion-transport function. This certainly represents a critical question considering the severe complication of alveolar instability generated by cyclic recruitment-derecruitment in edematous lung regions (Bates and Smith, 2018; Bates et al., 2020; Gaver et al., 2020). The impact of positive airway pressure is complex for many reasons, considering in particular that an opposite flow may occur, depending on alveolar pressure, across the endothelium and the epithelium. A ventilation strategy that would keep the acutely injured lung open and stable would theoretically be optimal at minimizing pulmonary edema accumulation. The critical issue remains to be minimizing tissue damage caused by lung overdistension, and at the same time, avoiding tissue injury by cyclic folding/unfolding.

## Author Contributions

EB, JG, GN, and GM joined their competences to complete the study. GM and EB conceived the work. FR and GS contributed to the computational part. All authors contributed to the article and approved the submitted version.

## Conflict of Interest

The authors declare that the research was conducted in the absence of any commercial or financial relationships that could be construed as a potential conflict of interest.

## Publisher’s Note

All claims expressed in this article are solely those of the authors and do not necessarily represent those of their affiliated organizations, or those of the publisher, the editors and the reviewers. Any product that may be evaluated in this article, or claim that may be made by its manufacturer, is not guaranteed or endorsed by the publisher.

## Footnotes

1
tcavnetwork.org
